# Leaving out the Patient

**Published:** 1917-02

**Authors:** 


### Leaving Out the Patient.

Dr. Robert T. Morris has an excellent article on this subject in the N. Y. Medical Journal for Nov. 4. He dwells on the unnecessary application of interesting scientific methods to cases, often entailing undue expense. We have been more impressed with the tendency on the part of some men to apply the methods of 1850 to the run of their private practice and to select some particular case of 1920 methods, to keep up their prestige. On the one hand, the average patient suffers from the lack of scientific investigation, on the other the exceptional one suffers from being assigned the role of a laboratory animal. In general, we are inclined to believe that the patient is left out of the consideration by the lack of scientific study and methods of treatment, more often than by letting science run to seed for the future instead of being brought to fruition for the immediate problem. But the general ethical problem involves many details. It is perhaps better to remind ourselves that the problem exists in every case, sometimes as a matter of business honesty, sometimes as a more general humanitarian issue, than to attempt to discuss all of its ramifications.

